# Domestic Animal Models of Central Nervous System Tumors: Focus on Meningiomas

**DOI:** 10.3390/life13122284

**Published:** 2023-11-30

**Authors:** Michele Tomanelli, Tullio Florio, Gabriela Coronel Vargas, Aldo Pagano, Paola Modesto

**Affiliations:** 1 Department of Experimental Medicine, University of Genova, 16132 Genova, Italy; gabrielafernanda.coronelvargas@edu.unige.it (G.C.V.); aldo.pagano@unige.it (A.P.); 2 Pharmacology Section, Department of Internal Medicine (DIMI), University of Genova, 16126 Genova, Italy; tullio.florio@unige.it; 3 IRCCS Ospedale Policlinico San Martino, 16132 Genova, Italy; 4 National Reference Center for Veterinary and Comparative Oncology, Veterinary Medical Research Institute for Piemonte, Liguria and Valle d’Aosta, 10154 Torino, Italy

**Keywords:** meningiomas, cancer, one health, veterinary medicine

## Abstract

Intracranial primary tumors (IPTs) are aggressive forms of malignancies that cause high mortality in both humans and domestic animals. Meningiomas are frequent adult IPTs in humans, dogs, and cats, and both benign and malignant forms cause a decrease in life quality and survival. Surgery is the primary therapeutic approach to treat meningiomas, but, in many cases, it is not resolutive. The chemotherapy and targeted therapy used to treat meningiomas also display low efficacy and many side effects. Therefore, it is essential to find novel pharmacological approaches to increase the spectrum of therapeutic options for meningiomas. This review analyzes the similarities between human and domestic animal (dogs and cats) meningiomas by evaluating the molecular and histological characteristics, diagnosis criteria, and treatment options and highlighting possible research areas to identify novel targets and pharmacological approaches, which are useful for the diagnosis and therapy of this neoplasia to be used in human and veterinary medicine.

## 1. Introduction

Intracranial primary tumors (IPTs) constitute a heterogeneous class of neoplasms characterized by a high mortality rate in humans. According to the International Classification of Oncology Diseases by the World Health Organization (WHO), IPTs are classified into more than 100 different tumor types [1]. They can manifest at any age and include both malignant and non-malignant tumors. In infants and adolescents, malignant tumors are the most frequently diagnosed IPTs, occurring at a rate of 3.55 cases per 100,000 individuals. Instead, non-malignant forms are less frequently diagnosed, with an incidence of 2.60 cases per 100,000 individuals [2]. The most common malignant IPT in infants is glioma, whereas pituitary tumor stands out as the most frequently occurring non-malignant form. The mortality rates exhibit variability but, on the whole, signify a substantial cause of death within this population group. In adults, IPTs rank as the eighth most common cancer. Although non-malignant tumors are more common in adults, with an incidence of 16.33 cases per 100,000 individuals, than in children, malignant tumors reach a noteworthy incidence of 7.08/100,000 [3]. Additionally, in adults, gliomas represent the most diagnosed form among malignant IPTs, whereas meningiomas and pituitary tumors are the most frequent non-malignant forms.

IPTs have been associated with fatal outcomes in various animal species, with extensive research conducted in dogs and cats. The most frequently occurring IPTs in dogs and cats include gliomas, meningiomas, and choroid plexus tumors. The incidence of these tumors appears to be higher in dogs compared to cats: about 14.5 cases per 100,000 dogs and an estimated 3.5 cases per 100,000 cats [4,5]. The incidence is strongly correlated with the age of the animal, with IPTs occurring in middle-aged to older dogs and in older cats. Most reports indicate that meningioma diagnoses occur in dogs over 7 years of age and in cats over 9 years of age [6]. In juvenile dogs, intracranial tumors are more commonly identified as neuroepithelial tumors of glial, neuronal, or embryonal origin [4]. One study found a statistically significant linear relationship between age and body weight and the occurrence of IPT in dogs [7]. Although meningiomas are the most common IPTs in dogs, oligodendrogliomas and gliomas are also very frequent [4,8]. Feline meningiomas have the highest incidence with respect to other brain tumor forms [9]. Canine and feline IPT incidence also seems to depend on the breed [7,9,10,11]. A genetic predisposition to canine glioma exists, with the protein expression of TP53, MDM2, P21, AKT, PTEN, RB1, P16, MTOR, and MAPK correlated to the specific glioma subtype [12]; moreover, a genome-wide association study followed by regional mapping [13] identified single nucleotide variants in three neighboring genes, *DENR*, *CAMKK2*, and *P2RX7*, which are highly associated with glioma susceptibility [13]. Intracranial tumors can also manifest as secondary tumors in dogs, representing about 50% of all intracranial tumor diagnoses and exhibiting a higher frequency than primary tumors. These metastases primarily originate from hemangiosarcoma (about 30%), lymphoma (about 20%), and metastatic carcinomas (about 20%) [11]. Intracranial tumors account for 2.2% of all tumors diagnosed in cats. It has been reported that meningiomas are the most prevalent primary brain tumors, comprising 58.1%, while lymphomas are the most frequently occurring secondary tumors, accounting for 14.4%.

In this review, we report comparative aspects of the etiology, histology, and diagnosis of meningiomas in humans, dogs, and cats. Additionally, we delve into the molecular aspects that characterize tumors in these three species, along with therapeutic approaches in order to summarize the similarities and differences, which can be useful in the study of these pathologies under a “One Health” perspective.

Following the “One Health” approach, various animal model validations were carried out for numerous types of cancer, including CNS tumors, to improve the medical practices in both the human and veterinary fields [14].

The focus of this review on meningiomas stems from their significance; despite the benign nature of a large percentage of these tumors, their growth can lead to dementia and clinical symptoms, severely compromising the quality of life. Moreover, a high rate of relapse is present in about 20% of histologically benign meningiomas, even after a radical excision, determining a poor prognosis. In some cases, meningiomas are diagnosed in multiple forms that cannot be treated by surgery [15,16,17,18,19]. However, surgery is not always curative and, under specific conditions, may pose risks to health. Therefore, the exploration of alternative therapeutic approaches, leveraging insights from animal models, could provide novel valid options for this type of neoplasm.

The literature predominantly features animal models of meningiomas involving murine and domestic species. Nonetheless, studies on meningiomas have also been conducted in horses, cattle, and pigs [20]. Murine models of meningiomas have facilitated the examination of mutations that characterize human tumors through primary cultures and xenografts. These models permitted the evaluation of the role of stem cells in tumor biology [21,22,23,24]. The use of murine models, mainly Genetically Engineered Mice (GEM) and Patient-Derived Xenograft (PDX), in oncology is particularly important due to their wide availability and economic feasibility. However, translating data from these models to humans presents several limitations, as discussed in previous reviews by Xu, C. et al. (2019) and Cho, S.Y. et al. (2020) [25,26]. GEMs offer a valuable tool for studying the response to immunotherapy, due to their intact immune systems, and to observe the physiological tumor environment, but other studies presented variability in the clinical presentation caused by altered phenotypes [26,27]. On the other hand, PDX models reflect the cellular complexity and histological architecture of real tumors, but, unlike GEM, they lack immune system information. Consequently, they are not suitable for studying immunotherapy [26].

In general, manipulating murine models at the central nervous system (CNS) level poses challenges due to their small size. The most notable limitation in mouse models lies in tumor induction. Induced models lack many important features found in spontaneously occurring models [27,28]. Several naturally occurring inherited and acquired neurological diseases, including meningiomas, that occur in dogs and cats have analogous counterparts in humans [29]. The genetic and environmental characteristics of tumors have been studied, revealing several analogies with human cancer [30,31,32,33]. Moreover, due to the close sharing of the environment, domestic animals and humans are exposed to similar environmental and risk factors that promote the spontaneous onset of tumors. This condition allows the use of domestic animals not only as models but also as a sentinel to predict an environmental risk for humans [34]. Some cases of sentinel dogs are reported in the literature [18,19,20,21,22,23]. However, it is important to remark the different incidence of specific tumors between domestic animals and humans. This aspect suggests an important role of biological risk factors present in both species in the onset of tumors and not only environmental factors [35,36].

Domestic animal models overcome some limitations observed in murine models when studying tumors. Consequently, domestic animals have been recognized as suitable pre-clinical models for investigating various types of tumors, including brain tumors [4,28,29,37]. In this context, the use of domestic animals as a model for meningiomas may allow comparative studies on a larger size of samples with histological, molecular, and neuro-imaging aspects similar to humans within immunocompetent organisms.

Moreover, the validation of the canine model may have an additional advantage, considering that the analysis of the canine genome reported nucleotide sequences and rearrangements more similar to the human genome than that of rodents and the sharing of similar gene expression profiles [37,38].

At the same time, domestic animals also have limitations in their use as a model for human cancer studies. The use of domestic animals implies higher efforts and costs for sample collection compared to the mouse model. The heterogeneity of the tumors can be variable between different species, and it could be a disadvantage during the study of specific molecular pathways if no in-depth knowledge exists. The incidence of the tumors is different between humans and pets for some cancer types [39]. Another obstacle can be the ethics and oversights of possible trials with pets. Every kind of trial (even the use of collected specimens for research) need an informed consent process [40] and the emotional state of the owners could affect the decision. Moreover, the execution of additional diagnostics procedures or invasive exams could be not accepted by the owners [40].

Table 1 summarizes the advantages and disadvantages of using domestic animals as translational cancer models.

In 2004, the Canine Comparative Oncology and Genomics Consortium (CCOGC) was established, creating an extensive biospecimen repository of canine cancers and tissues. The consortium strengthened connections among veterinary oncologists, researchers, and clinicians and initiated non-clinical trials employing dogs for the development of novel paths for new cancer drugs [41].

Brain tumors in dogs and humans arise spontaneously with similar incidence [4]. The existence of species in which tumors arise spontaneously represents the best translational model. An important advantage of the comparative studies of CNS cancer in companion animals is determined by the possibility of evaluating the role of the blood–brain barrier (BBB) in the pharmacokinetics and pharmacodynamics of the drugs; in fact, the physical size and vascular biology of the BBB are similar in pets and humans [42,43,44].

When considering brain cancers, it is important to highlight that these tumors represent a low percentage of canine cancer diagnoses. Therefore, the number of dogs that can be recruited for studies is low [40]. Conducting potential clinical trials involving dogs may take approximately 1–3 years, a duration that may be financially challenging for small biotechnology companies to support.

To improve the employment of companion animals as translational models for brain cancer in humans, a “Comparative Brain Tumor Consortium” (CBTC) was organized. The CBTC includes the comparative oncology program and the neuro-oncology branch by the National Cancer Institute (NIH) in association with an external community of radiologists, neuro-oncologists, radiation oncologists, and surgeons. The functions of CBTC include the study of the pathology and molecular markers, drug discovery, tumor biology, and immunology. Moreover, CBTC tries to simplify the organization of clinical trials regarding the recruitment of animals [32].

## 2. Meningiomas

Meningiomas frequently occur in humans and domestic animals. Although they are mostly benign, they can also exhibit malignant characteristics. In humans, meningiomas are the most common type of IPT, accounting for approximately 40% of all brain cancers, with a median age of diagnosis at 67 years [45,46,47]. Meningiomas represent around 30% of all primary CNS tumors in adults but only 0.4–4.6% in pediatric patients, with a higher proportion observed in females [48].

In humans, the most frequent site of origin for meningiomas is the arachnoid cells in the dura mater. Additional sites include the arachnoid associated with cranial nerves or choroid plexus [49]. Despite being often diagnosed as benign lesions due to their histological features, these tumors can show a clinical behavior similar to malignant neoplasia, with a high recurrence rate and poor prognosis. The 10-year overall survival for non-malignant meningiomas is approximately 81.4%, whereas for malignant forms, it decreases to 57.1% [45,46].

Meningiomas are also diagnosed in canine and feline species displaying a strong relationship with age and breeds and accounting for from 22.3% up to 50% of all brain tumors in dogs [4,11,50] and 58% in cats [51]. In a comparative study between canine and feline meningiomas, Wada et al. (2020) highlighted the development of tumors at median ages of 11.7 years and 14.1 years, respectively. According to the same study, the canine breeds most affected were Miniature Dachshund, Toy Poodle, Beagle, Shetland Sheepdog, Labrador Retriever, Flat-coated Retriever, Shiba Inu, Jack Russel Terrier, Welsh Corgi, and mixed breed [52]. A Japanese study that analyzed data from 186 canine intracranial tumors showed a breed predisposition for meningiomas in Rough Collie, Golden Retriever, Miniature Schnauzer, and Scottish Terrier [53]. Other studies associated a frequent meningioma diagnosis with canine dolichocephalic breeds [7,54,55,56], while a female sex predisposition was not confirmed in more recent studies [11,57]. In cats, Domestic Shorthair seems to be the most predisposed breed to meningioma and no significant difference between sexes exists [57]. Adamo et al. (2003) reported a higher onset of meningiomas in Persian, Domestic Shorthair, and Domestic Longhair cats [6]. In dogs, meningiomas originate in the calvarium-adjacent region, involving the olfactory and frontal regions, cranial cavity, optic chiasm, and suprasellar and parasellar regions, although it has been rarely diagnosed in other regions [58,59]. The sites in which feline meningiomas arise are mainly the tela choroidea of the third ventricle, the supratentorial meninges, and less frequently the cerebellar meninges [51,60,61]. Conversely to what happens in dogs, multiple meningiomas are frequently found in cats (about 17% of all meningioma cases) [51,57,62,63]. Three theories have been proposed to explain the occurrence of multiple meningiomas: multicentric dural foci, metastasis by blood-borne spread, and metastasis via the cerebrospinal fluid. The first hypothesis seems most plausible considering the tumor’s histologically benign nature and the reports showing histological variants in the same patient [64]. However, more research is needed to confirm them.

In about 15% of cases in cats and 20% of cases in dogs, meningiomas are diagnosed in the presence of other neurological disorders [51,57], including depression, stupor, coma, ataxia, lethargy, inappetence, and anorexia in cats [51] and menace response deficits, other cranial nerve deficits, ataxia, and reduced postural reactions in dogs [65].

## 3. Pathogenesis

The factors contributing to the development of meningiomas in humans and domestic animals need to be further studied. Currently, several hypotheses and mechanisms have been proposed.

Ionizing radiation stands out as the primary environmental risk factor consistently linked to the development of meningiomas. Human exposure to ionizing radiation leads to a 6- to 10-fold incidence increase in this condition [66]. Furthermore, this heightened risk is notably evident among survivors of the atomic bombings in Hiroshima and Nagasaki, where a substantial increase in meningioma cases has been documented [67,68]. In addition to radiation exposure, occupational contact with herbicides and pesticides also appears to elevate the likelihood of developing meningiomas [69]. Moreover, obesity has been identified as a significant positive risk factor for tumor development, likely due to its association with chronic inflammation and the signaling of insulin or insulin-like growth factors [70,71,72,73,74].

Several receptors are over-expressed in meningiomas, particularly somatostatin receptors (SSTRs) and intracellular receptors for sexual steroid hormones, such as androgens, progesterone, and estrogen, suggesting a role for these systems in tumor pathogenesis [67,75,76,77,78]. Among SSTRs, although all the subtypes are expressed in meningiomas [79], SSTR2 expression was associated with a poor prognosis, while SSTR1 expression was associated with reduced incidence of relapses, with less strong evidence. Nevertheless, the in vitro activation of SSTRs demonstrates antiproliferative effects. This evidence led to the development of clinical trials exploring the use of selective agonists, such as octreotide and pasireotide, although, to date, no conclusive results have been reported [80]. Receptors for the steroidal hormones estrogen (ERα) and progesterone (PR) have been reported to be expressed in most meningiomas: ERα presence has been associated with increased proliferation and the development of high-grade tumors, while the high expression of PRs has been correlated with Grade 1 tumors according to the WHO grading system [81]. It is now assumed that PR expression alone represents a favorable prognostic factor in meningiomas, while its loss or the association with ER expression correlates with a worse clinical outcome. However, pharmacovigilance data indicated that the prolonged use of androgen receptor antagonists and/or progesterone receptor agonists (cyproterone acetate, nomegestrol acetate) results in increased meningioma incidence [82,83], making rather complex the prognostic or therapeutic evaluation of these receptors.

Feline and canine meningiomas also express PR [6,84]. Adamo et al. (2003) reported a high proportion of PRs and the absence of ERs in feline meningiomas. Furthermore, a high number of cells with PRs and a significantly lower number of cells with ERs in canine meningiomas were observed. According to this study, the proportion of PR-positive cells in canine benign meningiomas was >80%, while in malignant meningioma only 32% of cells were PR-positive; in cats, the percentages were >80% and 38%, respectively. In dogs, the number of PRs correlated to more aggressive progression (with nuclear pleomorphism, severe necrosis, and histological subtypes), while in cats, such a correlation was not observed [6].

In humans, meningiomas occur in several forms, sometimes associated with other syndromes. Table 2 shows the syndromes associated with high-frequency meningioma and the gene assumed to be involved.

Some of the syndromes listed in Table 2 have also been modeled in animals or have been observed in other species. A murine model was developed to accurately replicate the human *NF2*-related schwannoma phenotype, including the deficit in hearing and balance [98]. Mice with mutations in *PTC*, an orthologue of human *PTCH1*, develop many of the characteristics of Gorlin syndrome and exhibit a high incidence of rhabdomyosarcomas [99]. *Pten*^M3M4^ missense knock-in mutant mice present megalencephalic brains and elevated nuclear proteasome activity, also observed in patients with Cowden syndrome-related mutations in PTEN [100]. Multiple endocrine neoplasia type-I-like syndrome was reported in two male Domestic Shorthair cats that developed symmetric alopecia, insulin-resistant diabetes mellitus, and pituitary-dependent hyperadrenocorticism at 12 and 13 years of age [101] and in a crossbred 12-year-old male dog with abdominal enlargement, seborrhoea, and polypnea [102]. Nevertheless, the listed studies did not highlight associations with meningioma.

Few studies reported contemporaneous and unrelated neoplasms in 3–23% of dogs with IPTs, mainly in the thoracic or abdominal cavity [4,11,103].

As reported in a review by Motta et al. (2012), intracranial meningiomas in dogs as well as in cats have been diagnosed with concurrent neural (oligodendroglioma and meningioangiomatosis) [11,51,104,105] or extra-neural disorders (mucopolysaccharidosis type 1 and thymic lymphoma) [106,107]. Moreover, it has been reported that 13.9% of cats and 19% of dogs develop a meningioma in addition to another intracranial neoplasm [11,51,104,108].

In humans, the malignant forms of meningiomas increase the tumor cell invasion processes and the risk of metastasis is higher compared to non-malignant meningiomas [109]. Metastases developments are reported mainly in the lung, pleura, bone, and liver. In domestic animals, meningioma metastases have been described almost uniquely in dogs, and mainly pulmonary metastases were observed [110,111]. A study performed in cats highlighted skull osteolysis. The authors hypothesized that metastases could be responsible for osteolysis [112].

## 4. Histopathological Classification

The WHO classifies meningiomas in humans and domestic animals with similar criteria.

In humans, the WHO classifies meningiomas into 15 subtypes, reflecting a broad heterogeneity [1]. These are clustered into three groups, differentiated by their histological components. The first group is composed of benign forms classified in different variants: meningothelial, fibrous, and transitional, which are the most common forms. Psammomatous, angiomatous, microcystic, secretory, lymphoplasmacyte-rich, and metaplastic variants are also included in group 1, but their incidence is significantly lower. In group 2, three additional classes are clustered: atypical, choroid, and clear cell meningiomas. The third group encompasses anaplastic, papillary, and rhabdoid meningiomas, often diagnosed as a single histological type, but including different biological and oncological aspects associated with different documented genetic mutations [113,114].

In domestic animals, meningiomas are divided into subtypes, according to the morphological characteristics of the cells [5]. Initially, WHO classified domestic animal meningiomas into two categories: benign and malignant. The benign meningiomas included eight subtypes: meningotheliomatous, fibrous, transitional, psammomatous, angiomatous, papillary, granular cell, and myxoid. Malignant meningioma was classified as anaplastic. However, this classification presents some limitations, and considering the similarities between human and domestic animal meningiomas in pathological, immunological, molecular, and MRI aspects, an improved classification was defined. The benign meningioma classification must be used with caution as histological aspects sometimes lead to considering benign neoplasia that does not match with biological/oncological characteristics [1,115,116,117]. In a study on feline meningiomas, concurrent benign and malignant forms were diagnosed [108].

Dogs show a rare form of meningioma, known as cystic meningioma, characterized by cysts originating through tumoral processes, such as necrosis or release of fluids. The size of the cyst depends on the fluid volume and causes increased intracranial pressure [118].

The observed similarities in pathological, immunological, and MRI aspects between human and canine meningiomas allowed for the classification of canine meningiomas according to three grades of the 2016 WHO human histological grading system [115]. In order to evaluate the possibility of translating the human grading system to canine tumors in terms of accuracy and reproducibility, Belluco et al. (2022) evaluated veterinary neuropathologists’ inter-observer agreement with the application of a human grading system to canine meningioma [115]. The reproducibility of each histologic criterion was evaluated to identify a possible disagreement. The authors proposed amendments to increase reproducibility in canine meningioma [115]. In their study, Belluco et al. (2022) proposed a criterion for canine meningioma classification (Table 3) based on mitotic grade in a specific area (2.37 mm^2^) of tumor tissue [115].

Commonly, canine meningiomas present characteristics similar to the group 1 WHO classification of human meningiomas. The group 1 meningioma subtypes include meningotheliomatous, fibrous (fibroblastic), transitional (mixed), psammomatous, angiomatous (angioblastic), papillary, granular cell, myxoid, and anaplastic (malignant) [119]. Canine meningiomas usually display transitional, meningothelial, microcystic, and psammonas histological aspects [20]. In some cases, canine meningiomas present chondroid, osseous, myxoid, and xanthomatous-like areas in meningotheliomatous and transitional subtypes. Another aspect highlighted in meningotheliomatous and transitional subtypes was polymorphic infiltration with or without tumor cells necrosis area. Feline meningiomas are commonly classified into the transitional and fibroblastic subtypes. However, feline meningioma histology is cytologically bland and uniform; for this reason, it is very difficult to adapt to the human WHO guidelines [20]. The Comparative Brain Tumors Consortium (CBTC) tried to establish the translational aspects of canine brain tumors as a model for their human counterparts [120]. The work of CBTC provided the foundation for a histologic atlas of canine glioma that included astrocytoma, oligodendroglioma, and undefined glioma [121]. The samples were collected from several institutions and analyzed with immunohistochemistry to evaluate the expression of specific markers [121]. Other criteria studied were infiltrations, necrosis, mitosis, and vascularization. A grading classification was built reporting these criteria [121]. The grading was compared with human tumor counterparts. We suggest repeating the same studies for meningioma. Belluco et al. (2022) evaluated the reproducibility of criteria used in the human meningioma grading when applied to canine meningioma [115].

## 5. Diagnosis

Meningiomas are diagnosed in both humans and domestic animals through a combination of clinical evaluations, imaging techniques, and histopathological analysis. The primary diagnostic tools employed to assess the presence of meningiomas are magnetic resonance imaging (MRI), computed tomography (CT), and positron emission tomography (PET) [122,123].

Specific conditions such as edema, cyst formation, change in vascularity, and necrosis are well detected by MRI [124], and novel imaging techniques are improving the diagnosis of brain lesions. The use of MRI contrast agents permits the distinction of the tumor from the normal tissues. Furthermore, neurosurgeons can use MRI to minimize the size of craniotomy, maximize tumor removal, and minimize damage to the surrounding brain. Moreover, diffusion-weighted imaging, magnetic resonance spectroscopy, and dynamic contrast-enhanced MRI are entering clinical practice [125,126,127,128,129], whereas cerebrospinal fluid (CSF) analysis can provide clinical parameters, such as altered protein content and leukocyte count, useful for diagnosing pathology [130]. Motta et al. (2012) reported the main MRI characteristics of dog and cat meningiomas [57]. Table 4 summarizes meningioma MRI features observed in canine, feline, and human meningiomas [128,131,132,133].

Immunohistochemistry (IHC) plays a significant role in the diagnosis of meningiomas. IHC can be performed in human, canine, and feline meningiomas, using similar markers (Table 5). Saito et al. (2021) described about 39 cases of feline meningiomas of various grades in which specific markers were observed to classify the tumors [134]. The study analyzed markers such as Cytokeratin, Vimentin, E-cadherin, β-catenin, N-cadherin, and Ki-67. In feline meningiomas, Cytokeratin was only recognized in particular histological phenotypes (fibrous and transitional types) and showed high immunoreactivity in some studies involving dogs and humans [134]. Vimentin tested positive in some cases of feline meningiomas. According to Saito et al., E-cadherin remained stable in all subtypes of meningiomas, making it a reliable marker for feline meningiomas. In some studies, N-cadherin was detected in many human and canine meningiomas [135,136]. β-catenin was analyzed in both canine and feline meningiomas. In feline meningiomas, while β-catenin was detected in over half of the analyzed meningiomas, its translocation to the nucleus (indicating the active form) was observed only in specific tumor types; in particular, translocation was not evaluated in atypical and anaplastic subtypes. In dogs, β-catenin was detected at the nuclear level, mainly in anaplastic meningiomas [137]. Ki-67 is an important proliferation index associated with many tumors, as well as in meningioma [138]. In the human literature, KI-67 expression correlates with tumor aggressiveness in meningiomas [139,140,141,142]. Matiasek et al. (2009) evaluated the role of KI-67 in dog meningiomas [143] considering about 70 canine meningiomas. The samples were analyzed via immunohistochemistry and 64 cases tested positive for KI-67 [143]. Some studies reported KI-67 be predictive for the survival of dogs with non-nervous tissue tumors [144,145,146,147]. Matiasek et al. (2009) did not find the same prediction; however, this was a retrospective study performed with a small sample size [143]. Janssen et al. (2023) studied the expression of KI-67 in 68 canine meningiomas to correlate KI-67 expression with the WHO grading of meningioma [148]. Many samples positive for KI-67 were classified as WHO grade I and the authors hypothesized a possible role of KI-67 in meningioma development, in particular during the early stage of these tumors [148]. Saito et al. (2021) did not correlate Ki-67 with specific subtypes of feline meningiomas [134].

Therefore, the above-mentioned observations highlight common characteristics in meningioma classification and similar markers in IHC among the three species.

## 6. Molecular Characteristics

Meningiomas shows numerous molecular alterations in both human and domestic animal tumors, and some similarities and differences have been reported.

In humans, many genetic alterations have been associated with meningioma development. Deletions of *NF2* seem to be a common characteristic condition of meningiomas [150]. Indeed, alterations in *NF2* have been recognized as meningioma’s driver mutation, being present in about 50% of sporadic meningiomas [150]. Neurofibromatosis type II determines the inactivation of merlin gene *NF2*, a tumor suppressor involved in cytoskeleton dynamics, tumor-associated increased motility, and the regulation of cell proliferation [151]. Another pivotal player in meningioma’s development is *EPB41L3*, also known as *DAL-1* or *4.1B*, a tumor-suppressor gene that encodes erythrocyte membrane protein-band 4.1-like 3, involved in cell–cell interaction and having an important role in the control of motility [152,153,154]. Decreased *EPB41L3* expression is observed in about 70% of meningiomas.

Alterations in chromosome 1 were frequently found in meningiomas [113,155] and include mutations in *TP73*, *CDKN2C*, *RAD54*, *EPB41*, *GADD45A*, and *ALPL* [156,157,158,159].

Loss of function in chromosome 14 is commonly found in high-grade meningiomas and includes inactivation in NDRG family protein 2 and maternally expressed gene 3 (*MEG3*), which were associated with a poor prognosis [113,160,161]

Loss of function in chromosome 9 due to the deletions of the cyclin-dependent kinase inhibitors 2A (*CDKN2A*) and 2B (*CDKN2B*) has been associated with the progression from Grade 2 to anaplastic meningioma (Grade 3) [113,162].

Whole-genome sequencing approaches have identified mutations occurring in *TRAF7*, *AKT1*, *KLF4*, *PIK3CA*, and *SMO*, although these mutations seem to be mutually exclusive with those associated with *NRF-2* [163,164,165].

*TRAF7* is mutated in about 20% of all meningiomas and is frequently associated with *KLF4*, *AKT1*, and *PIK3CA* mutations [113,164].

*AKT1* mutations are found in about 12% of Grade 1 meningiomas and, although less frequently, in Grade 2 and 3. Interestingly, about 50% of all *AKT1*-mutated meningiomas also show alterations in *TRAF7* [113,164,166]. *PIK3CA* mutations occur in about 7% of all meningiomas; they are mutually exclusive with *NF2* and *AKT1* and often associated with *TRAF7* mutations [163]. Other somatic mutations associated with meningiomas include *BAP1*, *SMARCB1*, *SMARCE1*, *BRAF-V600E*, *NOTCH2*, *CHEK2*, *PTEN*, *CDKN2A*, *CDKN2B*, and *DMD* [92,167,168,169,170,171,172,173,174].

In humans, telomerase alterations are frequently associated with an increased risk of meningioma development [175]. Telomerase mutations occur in all grades of meningiomas, the frequency being associated with the tumor grade [176,177,178]. The main somatic mutations occur in the promoter region at two specific hotspots, C228T and C250T, resulting in up-regulation of the protein and the increased survival of cancer cells [177]. Slavik et al. (2022) performed RNA-seq in 64 meningiomas to identify novel prognostic markers for these tumors. This study found the dysregulation of many transcripts involved in the WNT signaling pathway, highlighting the importance of the WNT pathway in meningioma development, as already reported for dogs [179].

Initially, meningiomas were studied in athymic nude mouse models, after PDX meningioma cell inoculation. The study by Rath et al. (2011) demonstrated that meningioma PDX retained the characteristics of the original tumors and that a stabilized cell line of meningiomas exhibited similar features to the tumors of origin [180]. A limitation of the xenograft models is the absence of the tumor microenvironment, which restricts the study of the aspects of meningiomas, including drug resistance. GEM overcomes this limit by having an intact tumor microenvironment. Peyre et al. (2018) utilized a model of transgenic mice in which alterations in *NF-2* and *CDKN2AB* were induced [181]. Nevertheless, as above reported, studying meningiomas in mice models does not yield the same results obtained with spontaneous animal models and, in general, the existence of spontaneous models is a fundamental tool for studying cancer and other pathologies. Indeed, they mimic key aspects in the investigation of novel therapeutical approaches that are absent in non-spontaneous models, i.e., the role of the microenvironment and of the immune system [182].

Few available studies explored the genetic factors involved in the formation of canine and feline meningiomas. However, canine and feline models are reported to be strong translational models for CNS diseases, like stroke, epilepsy, movement disorders, lysosomal storage diseases, Alzheimer’s and cognitive disorders, and neuro-oncology [29]. Differential expression regulation in orthologue genes of Homo sapiens in canine and feline meningiomas has been reported (Table 6). Partridge et al. (2020) highlighted the advantages of using canine and feline models to study meningiomas since the larger size of the animals allows for surgical handling of the CNS; moreover, the presence of an intact immune system and the molecular, histological, and neuroimaging characteristics make them comparable to human neoplasia [29].

Besides genetics, it is important to consider the role of epigenetics in meningioma development. Many studies aimed at the identification of specific methylation profiles that could explain the mechanisms of oncogenesis, and some have demonstrated an epigenetic role in human meningioma onset [113,190,191]. These epigenetics mechanisms include DNA methylation, defective chromatin remodeling, alterations in microRNAs, and the hypermethylation of *TIMP3*. Alterations in the methylation of the *TIMP3* promoter determine inhibition of the metalloproteinases and can be associated with a poor prognosis [113,192,193]. *TP73* inactivation by hypermethylation has been investigated as a possible risk factor for malignant meningiomas [192,194]. Interestingly, experimental evidence suggests that methylation profiles might be suitable to predict the clinical outcome of patients. Indeed, some studies showed that an altered methylation profile is associated with a worse prognosis [178].

Few studies analyzed the molecular alterations driving canine and feline meningioma pathogenesis [189]. Different genes resulted in down-regulation, for instance *MYOC*, *ALP*, *PRKD1*, *FHL5*, *TIE1*, *MCAM* and *PECAM1*. Courtay-Cahen et al. (2008) highlighted the following alterations in canine meningiomas: histone acetyltransferase p300, PDGF-β, thioredoxin reductase 1, mutS homolog 2 and 6, Dal-1, Clusterin-like 1 (Retinal), B-cell lymphoma, T-cell differentiation protein, BCL-2-like 11, IL-1α, and IL-1β [195]. An RNA-seq transcriptome analysis performed in canine meningioma determined a series of over-expressed genes, for example, *FOSB*, *KLF5*, *WNT*, *GEM*, *EGR1*, and *DACT2* [189]. The entire altered gene list is reported in Table 6 and includes also genes altered in both humans and dogs: *FOS*, *KLF5*, *BMPR1B*, *NR4A1*, *WNT5A*, *PDPN*, *MYRF*, *MYC*, *ALP*, *COL14A1*, *THSB1*, *MMRN2*, *FHL5*, *SFRP1*, *MCAM*, *DAAM2*, *AQP1*, *KITLG*, *PECAM1*, *OLFML3*, *ADCY2*, *NOTCH3*, *FBLN2*, *APOD*, *PTCH2*, *IGFBP6*, *GAS6*, *COL16A1*, *PIK3R1*, and *FMN2* [196,197,198,199,200,201,202,203,204,205,206,207,208,209,210,211,212,213,214,215,216,217,218,219,220,221,222]. A case-report study speculated about the relationship between mucopolysaccharidosis I (caused by alpha-L-iduronidase deletions) and the onset of meningiomas in cats [106].

The role of PR receptors in the pathogenesis of human and animal meningiomas has been already mentioned [6,84,223,224,225,226,227,228,229]. Other studies reported the important role of matrix metalloproteinases *MMP-2* and *MMP-9* in canine and human meningiomas, where they are involved in extracellular matrix degradation, required for tumor progression and recurrence [230,231,232,233]. Mandara et al. (2007) evaluated the expression of *MMP-2* and *MMP-9* in canine meningiomas, establishing correlations with *TIMP-1* and *TIMP-2* expression [234]. The authors found that *TIMP-1* levels were elevated in Grade 1 and Grade 2 meningiomas, but not in Grade 3 cases and hypothesized different pathways in which *TIMP-1* can be involved in the progression of meningiomas [174]. Cyclooxygenase-2 (*COX-2*) is reported to have an impact on canine meningiomas [235] and is over-expressed in some feline meningiomas [236]. However, only in humans, *COX-2* seems to be correlated with meningioma grade and local invasion [29] and is a marker used to classify the tumors [237]. Vascular Endothelial Growth Factor (*VEGF*) has been proposed as a prognostic marker in canine, where it seems to be inversely correlated with survival time [29], while in human meningiomas, it is a tumor recurrence marker [238]. The role of E-cadherin, N-cadherin, β-catenin and doublecortin (*DCX*) have been evaluated in both canine and human meningiomas. In canine tumors, N-cadherin, β-catenin, and *DCX* seem to have a positive correlation with invasion and anaplastic subtypes of meningiomas, instead in human meningiomas, it has not demonstrated a correlation and this role is still debated [29,136,239,240]. Alterations of *NF-2* in chromosome 22 do not seem to play an important role in the onset of canine meningiomas [183], as opposed to human meningiomas, where they have been reported to predispose to the development of the tumors [239].

Glucose transporter (Glut-1) is expressed at high levels in malignant meningiomas both in dogs and humans [29]. Boozer et al. (2012) evaluated the immunological infiltrate in canine meningiomas [241], reporting a prevalence of CD18+ microglia and macrophages surrounding and infiltrating the tumors; CD11d+ cells were also present. Lymphocyte infiltrate included mainly CD3+ T-cells and a sparse number of CD79a+ B-cells. In human meningiomas, similar T-cell and B-cell infiltrate have been found, but in both cases, the biological role of this infiltrate was not determined [242,243,244,245]. The proteins altered in human and dog meningiomas are reported in Table 7.

## 7. Role of Mitochondria in Meningiomas

The human mitochondrial DNA (mtDNA) genome counts 16,569 bp and is characterized by a high copy number per cell. This genome is constituted of 37 genes, including 13 genes encoding proteins involved in the oxidative phosphorylation system, 2 rRNA, and 22 tRNAs. Noncoding sequences are present in mtDNA only in a small segment called D-loop, which is characterized by regulatory elements for replication and transcription of mtDNA. Point mutations, insertions, deletions, and nucleotide substitution in D-loop have been associated to the development of some tumors [246,247,248] including brain neoplasia [249,250,251]. MtDNA is especially prone to mutations because mitochondria are the major site of the reactive oxygen species (ROS) production; therefore, mtDNA is constantly exposed to their mutagenic activity. In CNS tumors, including meningiomas, a considerable number of mutations have been found in mtDNA [251]. Moreover, alterations in levels of complex II and IV of the respiratory chain involved in oxidative phosphorylation were highlighted in meningiomas. These anomalies seem to be linked to *HIF-1α* deregulation [252].

The role of p53 in the regulation of mitochondrial respiration is well-known [253]. Being *TP53* mutated with high frequency in meningiomas, its involvement in mitochondrial dysfunction could be a key event [253,254]. In many cancer types, a frequent mtDNA molecular alteration is the deletion between nucleotides 8470 and 13447, called mtDNA^4977^. This mutation can alter the oxidative phosphorylation and biogenesis processes. A case study highlighted the frequent presence of this mutation in patients with meningiomas [250]. There is currently no available research regarding molecular modifications of mtDNA in canine and feline meningioma, but several studies have been performed in other tumor types and disorders, also of the CNS [255,256,257,258].

## 8. Role of miRNA in Meningiomas

MicroRNAs (miRNAs) are short non-coding RNA of about 22 nucleotides, distributed in the genome to modulate protein expression, by inducing the degradation/instability of the target mRNA or by inhibiting its translation. MiRNAs can interact with the 3′UTR—sequence or directly with mRNA causing protein translation arrest or mRNA degradation [259,260,261,262]. MiRNA deregulation has been demonstrated to have a role in tumor cell growth, proliferation, and migration in many cancers, including meningiomas. In the last 20 years, many studies have been focused on the identification of miRNAs playing a function in cancer [259,260,262,263,264,265,266,267]. Nowadays, miRNAs have been proposed as molecular biomarkers for diagnosis and prognosis in meningiomas. Considering type 1 and type 2 human meningiomas, an important role of miR-21, miR-34a, miR-143, miR-193b, miR-218, and miR-451 deregulation compared to normal tissues have been highlighted [268]. Kopkova et al. (2019) analyzed CSF samples from meningioma patients and found high expression of miR-196a-5p, miR-140-5p, miR-10a-5p in neoplastic tissues [269]. Zhi et al. (2016) studied a cohort of Chinese patients to identify circulating miRNAs as novel possible biomarkers for meningiomas, identifying deregulation of miR-106a-5p, miR-219-5p, miR-375, miR-409-3p miR-197, and miR-224 [270]. Slavik et al. (2020) demonstrated that miR-15a, miR-146a-5p, and miR-331-3p expression in meningiomas was correlated to the activity of important intracellular pathways such as NF-kB, VEGF, STAT3, PTEN/AKT/mTOR [271].

The main miRNAs involved in meningioma biology are summarized in Table 8.

The identification of miRNAs biomarkers in meningiomas could represent an important advancement also in the veterinary field. The diagnostic or prognostic role of miRNAs has been investigated in different animal tumors, as well as in meningioma. Foiani et al. (2021) studied a panel of 14 miRNAs, with high sequence identity between human and canine, in 41 canine meningioma biopsy samples [277]. Five miRNAs resulted down-regulated (miR-96, miR-145, miR-200a, miR-29c and miR-335), while three were up-regulated (miR-136, miR-146a and miR-155) when compared to normal arachnoid tissue. Some of the miRNAs listed in Table 8 play a role in canine and feline neoplasms, also. In particular, the role of miRNAs was highlighted in mammary gland tumors, melanomas, osteosarcomas, urothelial carcinomas, leukemia, hemangiosarcomas [278,279,280,281]. For a detailed review see Varvil and dos Santos (2023) [282]. The miRNAs listed in Table 8, which have been investigated in animal neoplasms are reported in Table 9. MiRNAs’ involvement in IPTs is a little explored field in veterinary medicine and in the future could be important to study their role also in these types of tumors.

## 9. Therapeutic Approaches

Therapeutic strategies to treat meningiomas in humans and domestic animals depend on the grade of advancement of the tumors.

The basic therapeutic strategy for meningiomas is surgery to remove the tumor and surrounding tissues to make sure that all the cancer cells are eliminated. In some cases, surgery is sufficient to eradicate the disease, but in others further treatment is needed. Surgery is classified according to the Simpson grading, from 1 to 5 [303]. This classification helps to predict an eventual recurrence at 10 years of overall free survival. Grade 1 is determined by complete tumor removal, including the resection of the underlying bone and associated dura. The symptomatic recurrence at 10 years is about 9% [303]. Grade 2 is characterized by complete removal associated with coagulation of dural attachment. Symptomatic recurrence at 10 years is estimated at around 19% [303]. Grade 3 is associated with complete tumor removal without resection of dura or coagulation. In these cases, symptomatic recurrence at 10 years is about 29% [303]. Grade 4 is associated with subtotal resection; symptomatic recurrence exceeds 40% [303]. Grade 5 is characterized by simple decompression with or without biopsy. These cases have a 100% of recurrence risk at 10 years [303,304,305,306].

Radiation therapy is used as a first-line option for meningiomas characterized by the involvement of nerve sheath or the cavernous sinus [307]. Stereotactic radiotherapy provides significant benefits in patients, increasing 5-year survival [308]. Brachytherapy and radionuclide therapy are used in the treatment of atypical and malignant meningiomas, providing in few studies advantages in the survival [308,309,310].

Although several studies showed limited efficacy, systemic therapies have been proposed as alternative approaches to treat meningiomas [311,312,313,314,315]. In this context, the availability of novel pharmacological agents is a priority. Drugs used for systemic therapy include: temozolomide, bevacizumab, irinotecan, everolimus, sunitinib, mifepristone, imatinib, doxorubicin, and vincristine. An important randomized, multicentric study (EORTC-1320-BTG) is ongoing to evaluate the efficacy in meningiomas of trabectedin, an inhibitor of active transcription acting on RNA polymerase II [316].

Since molecular alterations of NF2 are frequent in meningiomas, molecular pathways NF2-linked have been studied as possible targets for pharmacotherapy [150,317,318,319]. In particular, the role of merlin, whose function is related to the control of the mammalian target of the rapamycin (mTOR) pathway, has been considered, and in mouse models specific mTOR inhibitors (temsirolimus and everolimus) suppress meningioma growth [312]. Other studies investigated the association of the inhibition of mTOR with the activity of other molecular targets. In this context, AZD2014, a dual-inhibitor of mTOR1 and 2, is currently in 2 phase-II trials which include the treatment of different forms of meningiomas [316].

Anti-angiogenic molecules, such as bevacizumab, have not demonstrated high efficacy in the treatment of meningiomas [320,321].

Massive parallel sequencing technologies increased the knowledge of meningioma genomic landscape, highlighted the heterogeneity of these tumors and opening novel scenarios of possible target therapy in the future [166,316]. Researchers identified in the genome of a set of Grade 1 meningiomas, somatic copy-number alterations (SCNAs), rearrangements, mutations, and insertions and/or deletions in a total of 645 known cancer-associated genes. Several clinical trials are now testing the validity of compounds that target epigenetic alterations. In this field, a particularly promising compound is KDOAM 25, a KDM5 inhibitor [322,323,324]. KDM5 comprises a histone demethylase family that removes tri- and di-methylations of lysine form on histone H3. In a study conducted on myeloma KDOAM25, altered the methylation profile of the tumor, and increased cell-cycle arrest in G1 phase and the apoptotic processes [322]. Olar et al. (2017) studied the methylation profile of the patients to create predictions on the recurrence of the meningiomas. These criteria could add up to histological and clinical predictors [323].

In NF2 wild-type tumors, experimental drugs showed the ability to inhibit smoothened (SMO), a member of the hedgehog pathway and AKT1 [163,325,326]. Boetto et al. (2017) showed that SMO-mutated meningiomas had a higher recurrence rate with respect to AKT1 mutated-meningiomas and that, overall, SMO-mutated meningiomas had a poor prognosis as compared to AKT1-mutated ones [327]. An important phase II clinical trial is currently testing the efficacy of SMO, AKT1, and focal adhesion kinase (FAK) inhibitors in patients with malignant meningiomas [328].

FAK has synthetic lethality with NF-2 loss; therefore, Brastianos et al. (2023) tested GSK2256098 (FAK inhibitor) in NF-2 meningiomas, observing an increase in progression- free survival (PFS) in the presence of a good safety of the drug [328].

Considering the high levels of PD-L1 expressing cells in some meningiomas, in which correlated to poor prognosis, immunotherapy has been considered as treatment also for this neoplasia [329]. Grade 2 and Grade 3 meningiomas seem to have high levels of PDL-1, but the correlation of PDL-1 expression with treatment response is still not clear [330]. Garzon-Muvdi’s work illustrated five-clinical trials, conducted in the United States, starting in 2020 to evaluate the response of meningioma patients treated with the immune checkpoint inhibitor antibodies nivolumab, ipilimumab, pembrolizumab, and avelumab [330].

The role of anti-progesterone drugs in therapy is under investigation in human meningiomas [331,332,333]. The use of the progesterone antagonist RU486 has been evaluated in humans to treat meningiomas [332]. The results demonstrated a reduction in tumor size, which was evaluated through MRI analysis. Andersen et al. (2013) showed that the use of hormone therapy did not increase the risk of developing meningiomas, as speculated in other studies [333].

In dogs, treatment of choice for meningiomas is surgery, and the median overall survival time after excision is about 7 months [334]. Radiation therapy is another important approach to treat dogs. In some cases, the therapeutic protocols provide an association between radiotherapy and corticosteroids [184,334,335]. Radiotherapy, usually fractionated in 5 weeks [184,336] increases survival time with respect to dogs treated with surgery only, while the association between surgery and consolidation radiotherapy increases the survival of dogs with intracranial meningiomas of about 8 months [334]. Adjuvant radiation therapy is typically reserved for atypical or anaplastic meningiomas in a similar approach as in human patients [337].

In domestic animals, chemotherapy has been used with non-effective results. In particular, a study reported the comparative use of carmustine and lomustine treatment in association with corticosteroids and anticonvulsants [338]. Some case reports showed a low efficacy of alkylating agents in increasing dog survival as compared to corticosteroids and anticonvulsants alone [338].

Other studies reported the possibility of treating meningiomas with hydroxyurea [339,340], an antineoplastic drug frequently used in veterinary practice, for example for the treatment of leukemias or mast-cell tumors. Marconato et al. (2007) reported two cases of dog meningiomas treated with hydroxyurea, in which a survival increase of 4 months was observed [339]. Moreover, hydroxyurea treatment demonstrated efficacy in meningioma cell culture, showing a reduction in proliferation rate and cell-cycle arrest. However, in a veterinary case, the use of hydroxyurea was associated to dermatological side effects [339]. In another case report, hydroxyurea was used in combination with phenobarbital and prednisolone, but this drug association did not produce noteworthy results [341]. The combination between hydroxyurea and imatinib-mesylate showed effectiveness in dogs [342,343,344]. Imatinib-mesylate use in meningiomas derives from its inhibition of PDGFR, a tyrosine kinase receptor which plays a role in meningiomas progression. In humans, the association between these two drugs produced a prolonged survival as compared to imatinib alone and was well tolerated by the patients [343,344]. The same treatment was evaluated also in veterinary practice on Belgian Malinois dogs, causing a decrease in tumor size; however, the dogs died due to anesthesia issues, and results about survival and efficacy of the treatment are not available [342].

Gene therapy for meningiomas has been proposed in one study, but to date, the road to applying it in clinics appears still long [345].

The evidence that canine meningiomas express high levels of PR prompted to study the possibility of treating animals with antiprogestinic drugs [6].

Another tested approach was the possibility of adopting vaccinations that can increase the activation of the immune response toward meningiomas [346]. The vaccination involves the administration of peptides containing defined T-cell epitopes to stimulate immunity response. In this study, although it was evaluated the possibility of enhancing T-cell response to the tumor, a major B-cell activation was highlighted instead [346].

The gold-standard therapeutical approach for feline meningiomas is also represented by surgery [347,348,349] and the median survival after surgery is 26 months. It is worth highlighting that about 80% of the cats after surgical removal of the tumor, did not develop recurrence [63,347]. In a study of 17 cases, the treatment with radiotherapy after the removal of the tumor nodule has been proposed, but no evidence of better efficacy of this approach has been reported [347]. In general, cat meningiomas are treated with the same panel of drugs used in dogs. Yun et al. (2021) highlighted an advantage of the combination between hydroxyurea and prednisolone [350]. This additive effect allowed the reduction in the tumor size. Palliative therapies remain important for seizure control, edema, and intracranial pressure, using anti-epileptic drugs (phenobarbital and potassium bromide) and glucocorticoids (prednisone and dexamethasone). The effects of palliative therapies are studied to stabilize a therapeutic regimen [184,351,352].

## 10. Discussion

IPTs represent a heterogeneous group of malignancies that occur in both humans and domestic animals, such as dogs and cats [57,353]. These tumors develop in both malignant and non-malignant forms leading to a high mortality rate, mainly due to the lack of effective pharmacological approaches. Meningiomas are the most frequent forms of IPTs in humans, dogs, and cats. Although these tumors mainly occur in benign forms, several related syndromes, such as dementia, depression, stupor, coma, ataxia, lethargy, inappetence, and anorexia result in a significant reduction in the quality of life [16,354,355]. However, meningiomas can also assume malignant characteristics determining a poor prognosis. A recent study characterized a population of tumor stem-like cells regulated by chemokine receptors in a large series of human meningiomas, which were proposed as determinants of the development of the more malignant clinical course [24].

A number of inherited pathologies have been identified in dogs and cats, and many of these share similarities with human counterparts [30,31]. Various aspects, including phenotypes, genotypes, pathophysiology, progression and factors influencing disease onset exhibit similarities in human, canine, and feline diseases. These analogies enhance the potential for discovering novel therapeutical approaches [29,356,357]. Domestic animal models serve as valuable translational models for humans, as they share similar lifestyles, food intake, and environmental conditions. Furthermore, advancements in genomic, diagnostic, and molecular biology techniques have contributed to the enhancement of veterinary practice [32], facilitating comparisons with analogous disciplines commonly employed in the medical field.

In humans, meningioma diagnosis primarily relies on MRI techniques, with specific criteria established to classify the tumor’s developmental grade [358,359,360,361]. Surgery remains the optimal approach for treating these tumors across all three considered species, but it is not always curative. Clinical pharmacological strategies predominantly involve chemotherapy and targeted therapy [311,362,363,364,365,366]. Chemotherapy’s efficacy is limited and often accompanied by significant side effects, while targeted therapy presents a promising but relatively novel option, with numerous ongoing clinical studies. Despite extensive exploration of these aspects in human medicine, there is still significant potential for research in animal models [315,367,368,369,370]. A particularly intriguing area of investigation is the role of steroid hormones in meningioma pathogenesis. The high expression of ERα and PR has been linked to meningioma proliferation in both humans and domestic animals. Consequently, hormone therapy represents a crucial field for exploration, leveraging the observed similarities among species [229,371,372,373,374]. Additional research is still needed to elucidate the role of sex hormones, especially progesterone, and their receptors in both tumorigenesis and the progression of meningioma [375]. Saito et al. (2021) explored this aspect, but no correlation was found between castration or sterilization and meningiomas. However, it is worth noting a potential study bias due to the limited number of females considered [134]. Rzechorzek et al. (2019) suggested a potential protective role of estrogens in the onset of brain tumors and brain aging based on a study involving 281 dogs. The results confirmed the beneficial effects of estrogen on the brain [376]. Understanding this role could enable the utilization of estrogens in primary preventive, secondary preventive, and therapeutic strategies for meningiomas. The role of immunotherapy remains controversial, as the evaluation of PD-L1 levels in these tumors does not seem to correlate with a response to checkpoint inhibitors [329,377,378]. In this field, a successful strategy might be determined by the use of vaccination aimed at stimulating the activation of T-cells and B-cells against the tumor [346]. While gene therapy is a well-studied strategy studied for many tumors in clinical settings, it has not been extensively explored for meningiomas. Nonetheless, some works have speculated about its potential implementation in treating meningiomas in dogs and cat [379,380].

The genetics and molecular characteristics of human meningiomas are well understood, [113,181,317,381,382,383,384,385,386]. Gene expression profiles and molecular alterations have been more comprehensively studied in humans compared to dogs or cats. In this review, we report the primary molecular characteristics in the three species that reflect the current state of art in meningiomas. While not all the molecular pathways studied in humans have been explored in domestic animal meningiomas, preliminary studies have identified similarities in the molecular pathways underlying pathogenesis and in gene expression or alterations. These findings, combined with the observed similarities in genome sequences and molecular pathways among humans, dogs, and cats, as demonstrated in other studies and neoplasms, give hope for the identification of shared molecular alterations to focus on.

The role of mtDNA and miRNA in the pathogenesis and biological response of human meningiomas has recently been investigated. To date, alterations in mtDNA have not been assessed in canine meningioma. However, this aspect has been explored in other canine tumors and SNC disorders; therefore, future studies on meningioma in canines could provide novel insights, and knowledge developed in humans could serve as a promising starting point for veterinary research. The role of miRNA as biomarkers has been studied in various canine tumors, including meningiomas [248], but the field is more extensively explored in human tumors.

Another aspect to be explored in the veterinary field is the challenge in classifying and diagnosing animal meningiomas [387,388,389]. The WHO classification of meningiomas acknowledges similarities across the three species but also differences. The difficulty in identifying analogies between human and domestic animal subtypes, along with histological variations, could be attributed to a higher number of studies in humans compared to animals, or to less comprehensive histological investigations on meningiomas in the veterinary field. In this context, a future approach similar to that adopted by the Comparative Brain Tumor Consortium (CBTC) for the classification of canine glioma is desirable. The CBTC involved a panel of expert physician and veterinary neuropathologists to assess the features of canine glioma, determine the best classification from both human and veterinary perspectives, and establish a common ground at the most basic level of tumor diagnosis [120].

While surgical approaches and radiotherapy remain the primary treatments of choice, understanding the molecular alterations present in meningiomas across the three species could be valuable in assessing the potential use of specific targeted therapies, in addition to traditional approaches. Adopting a “One Health” approach and amalgamating aspects incompletely studied in one species compared to in others may contribute to completing the puzzle represented by issues related to diagnosis, classification, therapy, and the validation of prognostic markers. Such an integrated approach holds mutual benefit for both human and veterinary medicine in the fight against this pathology [14,390].

## 11. Conclusions

Meningiomas represent the most common non-malignant tumors of the central nervous system in humans, dogs, and cats, displaying comparable frequencies and molecular features. Despite their prevalence and the significant clinical challenges associated with meningioma in humans, dogs, and cats, alternative therapies have not been fully explored. Consequently, surgery remains the treatment of choice, albeit with potential disadvantages and adverse effects.

Here, we reported an overview of the current knowledge regarding meningiomas in humans, dogs, and cats with the goal of identifying potential similarities or discrepancies. The objective was to highlight areas that could benefit from a knowledge exchange facilitated by a “One Health” approach.

Specific molecular pathways have been highlighted, demonstrating their role in meningiomas across different species. These pathways, including *COX-2*, *PR*, *ER*, *MMP-2*, *MMP-9*, *GLUT-1*, *VEGF*, *FOS*, *KLF5*, *BMPR1B*, *NR4A1*, *WNT5A*, *PDPN*, *MYRF*, *MYC*, *ALP*, *COL14A1*, *THSB1*, *MMRN2*, *FHL5*, *SFRP1*, *MCAM*, *DAAM2*, *AQP1*, *KITLG*, *PECAM1*, *OLFML3*, *ADCY2*, *NOTCH3*, *FBLN2*, *APOD*, *PTCH2*, *IGFBP6*, *GAS6*, *COL16A1*, *PIK3R1*, and *FMN2*, could be assessed in the context of novel targeted therapies. Simultaneously, certain miRNAs (miR-200a, miR-335, miR-146a, miR-29c, miR-155, miR-96) could be further investigated as potential prognostic markers or therapeutic targets.

## Figures and Tables

**Table 1:** Advantages and disadvantages of using domestic animals instead of murine models as a model for human cancer.

Advantages	Disadvantages
Spontaneously occurring tumors	Higher effort and costs for sample collection compared to mouse model
Shared environmental and risk factors	Tumor heterogeneity between species
Intact immune system	Different incidence between human and pets
Similar clinical, molecular, histopathological, genetic characteristics	Ethics and oversights of trials with pets
Large size	Invasive exams could be needed
Similar diagnostic techniques	
Genome and involved pathways are very similar to those of humans	

**Table 2:** Syndromes associated with a considerable frequency of meningiomas.

Associated Syndromes	Meningioma Frequency	Associated Genes	References
Neurofibromatosis type 2, schwannomatosis, spinal tumors, spinal ependymoma	35%	*NF2*	[49,85,86]
Gorlin–Goltz syndrome	1–5%	*PTCH1*, *P53*	[87,88,89,90]
Cowden syndrome	8.25%	*PTEN*	[91]
BAP1 Tumor Predisposition Syndrome	<1%	*BAP-1*	[92]
Multiple Endocrine Neoplasia Type 1	-	*MEN1*	[93,94]
Werner syndrome	10.9%	*WRN*	[95]
Rubinstein–Taybi syndrome	Case report		[96,97]

**Table 3:** Canine meningioma classification criteria proposed by Belluco et al. (2022) [115].

Number of Mitoses (n)	Mitotic Grade
<4 mitoses in 2.37 mm^2^	Grade 1
4 ≤ n ≤ 20 in 2.37 mm^2^	Grade 2: tumors with sheeting architecture, small cells, hypercellularity, macronuclei and spontaneous necrosis
>20 mitoses in 2.37 mm^2^	Grade 3: high anaplasia

**Table 4:** Common MRI features observed in canine, feline, and human meningiomas.

MRI Features	Dog	Cats	Human
Margins	Defined	Defined	Irregular
Pre-contrast	Isodense to hyperdense (T1 and T2)	Isodense to hyperdense (T1 and T2)	Isodense to hyperdense (T1 and T2)
Contrast	Marked and uniform	Marked and uniform	Nonhomogeneous
Tumor associated edema	Mild	Mild	Severe
Hyperostosis	Rare	Frequent	Occasionally
Dura tail	Frequent	Frequent	Frequent
Mass observation	Correctly observed	Correctly observed	Correctly observed

**Table 5:** Immunohistochemical markers evaluated in human, canine and feline meningiomas.

Species	Markers	Reference
Human	Vimentin, KL-1, Claudin-1, NSE, S-100, EMA, Leu-7, Cytokeratin, N-Cadherin, KI-67	[149]
Dog	Vimentin, EMA, Claudin-1, CD18, CD1c, CD11d, CD3, IBA-1, Cytokeratin, β-Catenin, N-Cadherin, S100, Pancytokeratin, PGP9.5, CD34	[20,29]
Cat	Vimentin, E-Cadherin, β-Catenin	[134]

**Table 6:** Reported differentially expressed genes in canine and feline meningiomas.

Species	Gene Name	Reference
Dog	*CADM1* (also known as TSLC1)	[183]
Dog	*NF2*	[183]
Dog	*VEGF*	[143,184]
Dog	*MMP-2* and *MMP-9*	[185]
Cat	*MMP-2* and *MMP-9*	[185]
Dog	*SSTR2*	[186]
Dog	*PDGFR*	[187]
Dog	*FOLR1*	[188]
Dog	*FOSB*, *FOS*, *CTSE*, *ZC2HC1C*, *KLF5*, *BMPR1B*, *MYBL1*, *NR4A1*, *PAMR1*, *GJB2*, *GEM*, *UPK3B*, *EGR1*, *ZFP36*, *CHGA*, *NOL4*, *DACT2*, *AMPD3*, *MEDAG*, *KLKB1*, *TF*, *AMDHD1*, *GPR133*, *WNT5A*, *RIMS1*, *PDPN*, *COMP*, *LPAR3*, *NEAT1_2*, *STXBP6*, *MYRF*, *PERP*, *BMPER*, *IRF6*, *VWA5A*, *MYC*, *ERMP1*, *DMXL2*, *WFCD2*, *NEAT1_1*, *FAM210B*, *CTPS1*	[189]
Dog	*MYOC*, *ALP*, *CILP2*, *COL14A1*, *THBS1*, *MMRN2*, *ADAMTSL1*, *PRKD1*, *COL8A1*, *SPTBN5*, *FHL5*, *MYH11*, *SFRP1*, *SCN7A*, *ARHGEF15*, *FRZB*, *SYNPO2*, *ACTC*, *PTGIR*, *TIE1*, *CD93*, *MCAM*, *FLT1*, *EMCN*, *MYCT1*, *DAAM2*, *KANK3*, *LAMC3*, *CALCRL*, *CPZ*, *DCP1/ACE*, *AQP1*, *KITLG*, *TINAGL1*, *PECAM1*, *CRISPLD2*, *OLFML3*, *ESAM*, *ADCY2*, *NOTCH3*, *FBLN2*, *FAM180A*, *STC2*, *APOD*, *PTCH2*, *APOE*, *CACNA2D2*, *PTPRB*, *FAM198B*, *MGLL*, *PDGFRL*, *TEK*, *IGFBP6*, *GAS6*, *ABCA8*, *TLL1*, *COL16A1*, *PALD1*, *PIK3R1*, *FMN2*, *MCF2L*, *NOS3*, *ARAP3*, *FMOD*, *DYSF*, *CYYR1*, *PLVAP*, *DRP2*, *ZFHX4*, *ECE1*, *GM2A*, *RCN3*, *AMOT*, *LEPREL2*, *TM4SF18*, *KALRN*, *PREX2*, *HECW*, *CCDC80*, *LIMCH1*, *ETS1*, *MYO1E*, *SHE*	[189]

**Table 7:** Proteins frequently altered in human and dog Meningiomas.

Genes	Species	Reference
MMP-2	Human, Dog	[185]
MMP-9	Human, Dog	[185]
TIMP-1	Human, Dog	[185]
TIMP-2	Human, Dog	[185]
COX-2	Human	[208]
VEGF	Human	[211]
N-Cadherin	Dog	[212,213]
β-Catenin	Dog	[239,240]
DCX	Dog	[239,240]
NF-2	Human	[183]
Glut-1	Human, Dog	[29]
CD18+	Dog	[242,243,244,245]
CD11d+	Dog	[242,243,244,245]
CD3+	Dog	[242,243,244,245]
CD79a+	Dog	[242,243,244,245]
CD34	Dog	[4]
NSE	Dog	[4]
PGP9.5	Dog	[4]
Pancytokeratin	Dog	[4]
S100	Dog	[4]

**Table 8:** Differentially regulated miRNAs in human meningiomas from different tissues.

Cases-Studies	miRNA Up Regulated	miRNA Down Regulated	miRNA Status Not Specified	Reference
Grade I and II meningiomas biopsies	miR-34a, miR-218	miR-21, miR-143 miR-193b miR-451	not specified	[268]
Cerebrospinal fluid from meningioma patients	miR-10a-5p, miR-140-5p, miR-196a-5p, miR-196b-5p		not specified	[269]
Circulating miRNAs	miR-106a-5p, miR-219-5p, miR-375, miR-409-3p, miR-1275, miR-657, and miR-224	miR-107, miR-129-3p, miR-1285-3p, miR-197-3p	not specified	[270]
Variable Grade of Meningiomas patients	miR-146a-5p, miR-155-5p, miR-96-5p	not specified	miR-15a, miR-331-3p, miR-21a, miR-107, miR-137 and miR-29b, miR-29c, miR-200a, miR-335	[271,272,273]
Variable Grade of Meningiomas patients	not specified	not specified	miR-197, miR-34a, miR-375, miR-219a, miR-224, miR-21, miR-200a, miR-409	[274,275]
Grade I and grade III meningiomas biopsies	not specified	miR-195		[276]

**Table 9:** miRNAs differentially regulated in human meningiomas, which have a role in different canine malignancies.

miRNA	Function/Mutational Status	Reference
miR-cluster 14q32	Important role in c-myc regulation	[278,280,283]
miR-17-5p	Over-expressed in canine lymphomas	[278,284,285]
miR-21	Up-regulated in canine mammary tumors	[278,281,286,287,288]
miR-34	Deleted in canine mammary tumors	[278,289,290,291,292]
miR-124a	Role in apoptosis control in canine hemangiosarcoma	[278,293,294]
miR-145	Inhibits cell growth in melanoma cells	[277,278,295,296,297]
miR-200	Functions in EMT regulation	[277,278,288]
miR-203	Reduces cell proliferation in canine lymphoma cells	[278,289,296]
miR-205	Down-regulated in melanoma	[278,298,299]
miR-210	Over-expressed in canine mammary tumor	[278,300,301]
miR-221, miR-222	Up-regulated in canine prostate cancer	[278,302]
miR-96, miR-145, miR-200a, miR-29c miR-335	Down-regulated in canine meningioma	[277]
miR-136, miR-155, miR-146a	Up-regulated in canine meningioma	[277]

## Data Availability

Not applicable.
